# Binding Heterogeneity of Plasmodium falciparum to Engineered 3D Brain Microvessels Is Mediated by EPCR and ICAM-1

**DOI:** 10.1128/mBio.00420-19

**Published:** 2019-05-28

**Authors:** Maria Bernabeu, Celina Gunnarsson, Maria Vishnyakova, Caitlin C. Howard, Ryan J. Nagao, Marion Avril, Terrie E. Taylor, Karl B. Seydel, Ying Zheng, Joseph D. Smith

**Affiliations:** a Seattle Children’s Research Institute, Seattle, Washington, USA; b Department of Bioengineering, University of Washington, Seattle, Washington, USA; c Blantyre Malaria Project, University of Malawi College of Medicine, Blantyre, Malawi; d Department of Osteopathic Medical Specialties, College of Osteopathic Medicine, Michigan State University, East Lansing, Michigan, USA; e Center for Cardiovascular Biology, Institute for Stem Cell and Regenerative Medicine, University of Washington, Seattle, Washington, USA; f Department of Global Health, University of Washington, Seattle, Washington, USA; Washington University School of Medicine

**Keywords:** PfEMP1, *Plasmodium falciparum*, cerebral malaria, microvessels, tissue engineering

## Abstract

Cerebral malaria is a severe neurological complication associated with sequestration of Plasmodium falciparum-infected erythrocytes (IE) in the brain microvasculature, but the specific binding interactions remain under debate. Here, we have generated an engineered three-dimensional (3D) human brain endothelial microvessel model and studied P. falciparum binding under the large range of physiological flow velocities that occur in both health and disease. Perfusion assays on 3D microvessels reveal previously unappreciated phenotypic heterogeneity in parasite binding to tumor necrosis factor alpha (TNF-α)-activated brain endothelial cells. While clonal parasite lines expressing a group B P. falciparum erythrocyte membrane protein 1 (PfEMP1) present an increase in binding to activated 3D microvessels, P. falciparum*-*IE expressing DC8-PfEMP1 present a decrease in binding. The differential response to endothelium activation is mediated by surface expression changes of endothelial protein C receptor (EPCR) and intercellular adhesion molecule 1 (ICAM-1). These findings demonstrate heterogeneity in parasite binding and provide evidence for a parasite strategy to adapt to a changing microvascular environment during infection. The engineered 3D human brain microvessel model provides new mechanistic insight into parasite binding and opens opportunities for further studies on malaria pathogenesis and parasite-vessel interactions.

## Abstract (precis)

Cerebral malaria research has been hindered by the inaccessibility of the brain. Here, we have developed an engineered 3D human brain microvessel model that mimics the blood flow rates and architecture of small blood vessels to study how P. falciparum*-*infected human erythrocytes attach to brain endothelial cells. By studying parasite lines with different adhesive properties, we show that the malaria parasite binding rate is heterogeneous and strongly influenced by physiological differences in flow and whether the endothelium has been previously activated by TNF-α, a proinflammatory cytokine that is linked to malaria disease severity. We also show the importance of human EPCR and ICAM-1 in parasite binding. Our model sheds new light on how P. falciparum binds within brain microvessels and provides a powerful method for future investigations of recruitment of human brain pathogens to the blood vessel lining of the brain.

## INTRODUCTION

Cerebral malaria (CM) is a life-threatening complication of Plasmodium falciparum infection associated with extensive infected erythrocyte (IE) sequestration in the brain microcirculation (1), alterations in coagulation (2), and severe brain swelling in fatal pediatric cases (3). CM carries a high mortality rate of 15 to 20%, even in hospitalized patients that receive effective antimalarial drugs (4). Despite the importance of IE cytoadhesion in CM pathogenesis, there is still limited understanding of the host-receptor interactions responsible for parasite sequestration in the cerebral microcirculation, largely due to the lack of an adequate experimental animal model or an *in vitro* human brain microvascular model.

Cytoadhesion of P. falciparum-IE is mediated by a multicopy gene family called *var*, which encodes P. falciparum erythrocyte membrane protein 1 (PfEMP1). Members of this protein family are expressed in a mutually exclusive fashion at parasite-induced, knob-like protrusions on the erythrocyte surface (5–7). PfEMP1 proteins contain multiple adhesion domains known as Duffy binding like (DBL) and cysteine-rich interdomain regions (CIDR). Individual domains bind to diverse receptors, including CD36, ICAM-1 (intercellular adhesion molecule 1), and EPCR (endothelial protein C receptor) (8). PfEMP1 proteins have diverged in binding properties, such that group A and DC8 proteins bind to EPCR (CIDRα1 domains) (9, 10), while group B and C proteins bind to CD36 (CIDRα2 to -6 domains) (11, 12). In addition, the ICAM-1 binding subset has diverged into PfEMP1 with dual binding activity for EPCR and ICAM-1 (found in group A) (13, 14) or CD36 and ICAM-1 (found in group B) (15, 16). Parasite tropism for different microvascular beds is likely to be influenced by host receptor expression levels and the multivalent binding properties of the expressed PfEMP1 variant, as well as microvessel parameters, such as lumen dimensions, branching architecture, and microfluidic properties. However, these parameters are not accurately modeled in two-dimensional models.

Severe malaria patients present widespread IE sequestration in multiple endothelial beds (17). Whereas multiple studies in humans have shown that a population of parasites expressing EPCR-binding DC8 and group A *var* genes is expanded in the peripheral blood of severe malaria patients (18–22), there are still large gaps in our understanding of how parasites sequester in brain microvessels. A recent study has revealed that IE from CM patients presented higher binding rates to brain endothelial cells than IE from uncomplicated malaria (UM) patients (23). Furthermore, group A and DC8-PfEMP1 variants have affinity for brain endothelial monolayers *in vitro* (24, 25), and IE preferentially sequester on ICAM-1-positive brain microvessels in CM autopsies (26). Taken together, these findings implicate the P. falciparum EPCR and ICAM-1 binding phenotypes in CM.

Malaria infection is accompanied by widespread endothelial activation, including changes in expression levels of both EPCR and ICAM-1 (26, 27). However, there has been limited investigation of whether parasites sense receptor differences between resting and activated endothelium and how parasite binding heterogeneity influences vascular interactions. Recent advances in bioengineering have led to the development of simplified three-dimensional microvessel models, in which endothelial cells are grown in collagen scaffolds. Engineered 3D microvessels better reproduce the physiological nature of the microvasculature, such as vessel three-dimensional geometry and appropriate pressure and flow characteristics, and provide suitable *in vivo*-mimicking models for the study of blood-endothelial interactions (28, 29). To investigate the P. falciparum-brain microvessel interactions under more physiological conditions, we adapted this technique to develop an engineered 3D human brain endothelial microvessel model (3D brain microvessels). By perfusing 3D brain microvessels with different P. falciparum binding variants, we have gained novel molecular insight into host receptor interactions that contribute to parasite binding and how endothelial activation influences parasite sequestration.

## RESULTS

### Development of a 3D human brain microvessel model.

**FIG 1:**
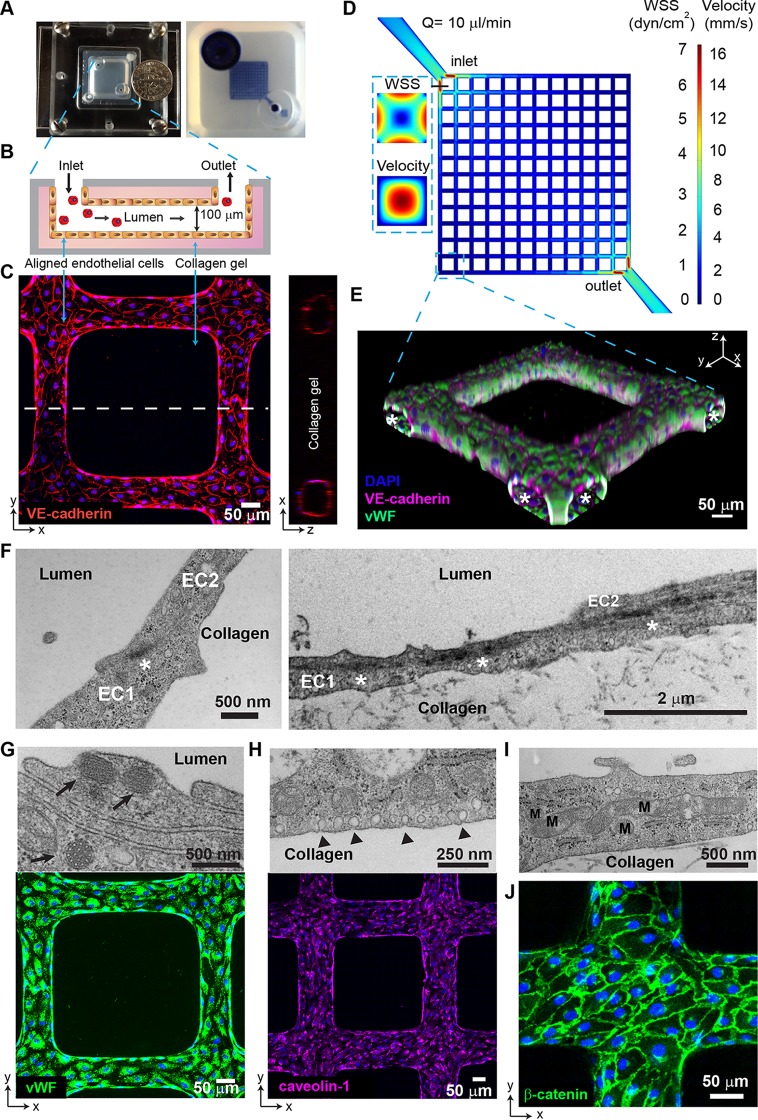
Characterization of 3D brain microvessels. (A) (Left) Photo of an assembled 3D microvessel device with a dime (diameter, 17.9 mm). (Right) 3D microvessels perfused with food dye. (B) Schematic cross-sectional view of the 3D microvessels. (C) Immunofluorescence assay (IFA) z-projection of confocal sections of a 3D brain microvessel (left) and cross-sectional view (right) labeled with anti-VE-cadherin (red) and DAPI (blue). (D) Mid-plane flow velocity (z = 50 μm) and estimated WSS (z = 0 μm) distributions in the grid geometry, simulated with COMSOL prior to collagen remodeling by HBMEC (see Materials and Methods). Inlaid cross-sectional views represent the lumen at the first branch after the inlet. (E) 3D reconstruction of a grid portion. Colors indicate anti-VE-cadherin antibody (red), anti-VWF antibody (green), and DAPI (blue). Asterisk, lumen. (F) Transmission electron microscopy (TEM) showing endothelial junctions and focal contacts. EC1 and EC2, endothelial cells 1 and 2; asterisk, electron-dense contacts. (G) TEM showing Weibel-Palade bodies (arrows, top) and IFA z-projection of VWF (green, bottom). (H) TEM image of polarized caveolae (arrowheads, top) and IFA z-projection of caveolin-1 (magenta, bottom). (I) TEM image reveals high mitochondrial (M) content of HBMEC. (J) IFA z-projection of adherens junctions stained with anti-β-catenin antibody (green). Nuclei in panels G, H, and J were stained with DAPI (blue).

To mimic the microfluidic properties of the brain microvascular plexus, we have engineered a 3D human brain microvessel model that presents a 13-by-13 grid network geometry, with a total of 312 branches, a diameter of 80 to 100 μm at each branch, and a total intravascular volume of approximately 1 μl (Fig. 1A to D). 3D microvessels present a similar surface-to-volume ratio (20 mm^2^/mm^3^) to small postcapillary venules (40 to 200 mm^2^/mm^3^). The scaffold was fabricated in collagen gel using injection molding and soft lithography techniques. The grid network design mimics the acceleration/deceleration microfluidic properties of a branching microcirculatory network and provides a large range of flow velocities and wall shear stresses (WSS) (i.e., 41-fold range comparing the maximal velocity near the inlet and the minimum near the opposite corner). After 3 days of culture, primary human brain microvascular endothelial cells (HBMEC) in these networks formed fully endothelialized lumens that can be perfused with different blood components, including P. falciparum-IE (Fig. 1B to E).

The 3D brain microvessels express endothelial markers, such as CD31 and the brain-enriched marker glucose transporter-1 (GLUT-1) (see Fig. S1 in the supplemental material). Ultrastructural imaging confirmed the presence of complex endothelial cell junctions and focal electron-dense contacts (Fig. 1F). 3D brain microvessels have several features characteristic of brain endothelial cells (30–33), including high expression of von Willebrand Factor (vWF) contained in Weibel-Palade bodies (Fig. 1G), a thin nonfenestrated cytoplasm not thicker than 2 μm (Fig. 1F and Fig. S1), caveolin-1 expression and vesicles on the microvessel luminal and abluminal sides (Fig. 1H), and a high number of mitochondria (total cellular area in transmission electron microscopy [TEM] sections of 12.2% ± 5.3%; 95% confidence interval [CI]), suggesting high cellular metabolism (Fig. 1I) (28, 34). Furthermore, 3D brain microvessels present a junctional localization of VE-cadherin (Fig. 1C) and β-catenin (Fig. 1J), demonstrating the presence of adherens junctions. Altogether, engineered 3D brain microvessels represent a unique perfusable human-based *in vitro* platform for the study of the IE-brain endothelium interaction at a wide range of flow velocities and WSS in a single device.

10.1128/mBio.00420-19.2 FIG S1 Additional characterization of 3D brain microvessels. The human brain microvascular endothelial cell (HBMEC) phenotype was confirmed by labeling with anti-CD31 (A) and anti-GLUT-1 (B) antibodies and IFA z-projection of confocal images. (C) TEM image showing cytoplasm thickness and the junction between two adjacent endothelial cells in 3D brain microvessels. EC1 and EC2, endothelial cells 1 and 2. Download FIG S1, TIF file, 1.4 MB. Copyright © 2019 Bernabeu et al. 2019 Bernabeu et al. This content is distributed under the terms of the Creative Commons Attribution 4.0 International license.

### P. falciparum flow-dependent binding in 3D brain microvessels.

**FIG 2:**
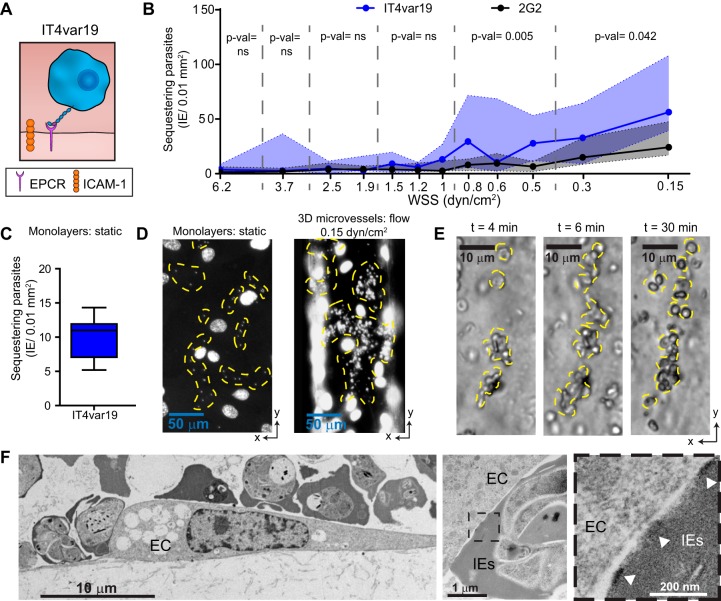
WSS-dependent binding of P. falciparum IE in 3D brain microvessels. (A) Cartoon schematic representation of IE expressing IT4var19 binding to EPCR. (B) Binding of IT4var19 and the control knobless 2G2 line at the different estimated WSS regions in the 3D microvessels. Medians are represented by dots and interquartile range by colored regions. Statistical analysis of binned regions (dotted lines) was determined by a Mann-Whitney test (*n* = 4 independent biological replicates) (see Fig. S3 and Materials and Methods for study design). (C) Binding of IT4var19 to resting HBMEC monolayers in static binding assays (*n* = 9 biological replicates). In the box plot, horizontal lines indicate medians, and whiskers show the 10th and 90th percentiles. (D) DAPI-stained representative images of IT4var19 binding to HBMEC monolayers (left) and 3D microvessels (right). (E) Time-lapse images showing a cluster of sequestered parasites growing over time. Sequestered parasites in panels D and E are highlighted with a yellow dashed outline. (F) Representative TEM images of IE cytoadhering to HBMEC (EC). Knobs are indicated with arrowheads.

The consequences of PfEMP1 phenotypic diversity for IE-vessel wall interactions are poorly understood. To investigate P. falciparum-IE-endothelium interactions in the 3D brain microvessel, we perfused IT4var19, a clonal parasite line that was previously selected *in vitro* on human brain endothelial monolayers and predominantly expresses a DC8-EPCR-binding PfEMP1 variant (*It4var19*) (Fig. 2A) (24, 35, 36). The predominant expression of the PfEMP1 of interest was confirmed by real-time PCR using *var* strain-specific primers (see Fig. S2 in the supplemental material). *In vivo* microcirculatory flow velocity and WSS are dynamic and vary in health and disease. Under healthy conditions, flow velocity ranges from 0.3 to 15 mm/s throughout the arteriovenous microcirculation, and WSS is estimated to range between 5 and 40 dyn/cm^2^ in small arterioles and capillaries and 1 and 5 dyn/cm^2^ in venules (37–39). However, lower WSS may be present under pathological conditions due to flow reductions and upstream vessel blockages (37). The range of flow velocities of IE within our model can be varied by adjusting the total inflow rate with a syringe infusion pump. When perfused at a flow rate of 10 μl/min, the 3D microvessel grid geometry reproduces physiological flow velocities, ranging from 0.3 to 16 mm/s and an estimated WSS of 0.15 to 6.2 dyn/cm^2^ (Materials and Methods) (Fig. 1D; see Movies S1 and S2 in the supplemental material), similar to flow velocities present in the arteriole-capillary-venule unit.

10.1128/mBio.00420-19.3 FIG S2 *var* architecture and transcription profile of P. falciparum lines. (A) Schematic representation of the PfEMP1 preferentially expressed by the P. falciparum parasite lines. Domain cassettes are indicated by lines and domain binding activity to endothelial receptors by arrows. The *var* gene transcription profile of ring-stage IE was analyzed by qRT-PCR with IT4 (B) (15) and HB3 (C) (57) *var* strain-specific primer sets. Transcription unit levels are normalized to the housekeeping control gene coding for STS (seryl-tRNA synthetase). nd, not determined. Download FIG S2, PDF file, 0.4 MB. Copyright © 2019 Bernabeu et al. 2019 Bernabeu et al. This content is distributed under the terms of the Creative Commons Attribution 4.0 International license.

10.1128/mBio.00420-19.8 MOVIE S1 Real-time video of IT4var19 perfusion (2 × 10^7^ IE/ml) at a low-velocity–low-WSS (0.15 dyn/cm^2^) region. Acquisition time is shown at the top left corner (*t* = 0 start of perfusion). Download Movie S1, AVI file, 8.9 MB. Copyright © 2019 Bernabeu et al. 2019 Bernabeu et al. This content is distributed under the terms of the Creative Commons Attribution 4.0 International license.

10.1128/mBio.00420-19.9 MOVIE S2 Real-time video of IT4var19 perfusion (2 × 10^7^ IE/ml) at a moderate-velocity–moderate-WSS (2.5 dyn/cm^2^) region. Acquisition time is shown at the top left corner (*t* = 0 start of perfusion). Download Movie S2, AVI file, 7.6 MB. Copyright © 2019 Bernabeu et al. 2019 Bernabeu et al. This content is distributed under the terms of the Creative Commons Attribution 4.0 International license.

10.1128/mBio.00420-19.4 FIG S3 Binding quantification strategy in the 3D brain microvessels. Schematic of the 13-by-13 microvessel grid design showing the location of the inlet and outlet. Arrows indicate the direction of flow throughout the grid network. (i) To avoid the effects of flow interdigitation, binding quantification was performed along columns 1 and 13, where flow either purely converges or diverges. Quantification of these columns gives an estimated WSS range from 0.15 dyn/cm^2^ to 6.2 dyn/cm^2^, with an overall ∼2-fold variation in WSS between branch points in the columns. (ii) Images of all branches were taken, and a z-stack of the bottom half of the vessel was created. Parasite binding was determined as the average binding in three squares (A, B, and C) that include the 80% central portion of the vessel. The 10% portion near the edges was excluded as WSS is less predictable in these regions due to edge effects, potentially leading to parasite trapping. The average of IE binding in squares A, B, and C was determined and expressed as IE/0.01 mm^2^. (iii) Representative z-stack image used for IE binding quantification. Scale bar, 50 μm. Download FIG S3, PDF file, 1.4 MB. Copyright © 2019 Bernabeu et al. 2019 Bernabeu et al. This content is distributed under the terms of the Creative Commons Attribution 4.0 International license.

To study the flow-dependent interaction of parasites and 3D brain microvessels, a solution of mature-stage P. falciparum was perfused for 30 min, followed by a 10-min wash to remove unbound parasites before fixation. A low-hematocrit P. falciparum-IE solution was used to avoid vessel clogging that could lead to alterations in predicted WSS and velocity. By visual inspection, none of the microvessel branches was completely obstructed or clogged by the endpoint of the perfusion, and flow was not interrupted at any branch. The parasite binding was determined at the endpoint, and the quantification strategy is summarized in Fig. S3 in the supplemental material. Of interest, the extent and pattern of binding differed between monolayer and engineered 3D brain microvessel platforms. Binding of IT4var19-IE (EPCR binder) was flow dependent, with the highest binding occurring at regions lower than 1 dyn/cm^2^ and the lowest binding at regions of high WSS (Fig. 2B). Notably, the median adhesion level of IT4var19 was 2.5 to 5 times higher at the lowest-WSS regions (<0.31 dyn/cm^2^) than in static binding assays (Fig. 2B and C). We also observed that IT4var19-IE display a distinctive concentrated binding pattern in 3D microvessels that started with a few IE and extends over time (Fig. 2D and E; see Movie S3 in the supplemental material). By comparison, a uniform scattered binding pattern is primarily found in static adhesion assays on HBMEC monolayers (Fig. 2D) (24). Ultrastructural analysis of 3D microvessels confirmed the high binding rate, showing multiple IT4var19-IE bound to a single HBMEC and demonstrating close contact between endothelial cells and IE via electron-dense knobs (Fig. 2F). A recent study has suggested that IT4var19 binding to an immortalized HBMEC line is inhibited by malaria-naive human serum, raising the possibility that DC8-EPCR interactions do not occur in human infections (40). However, binding of IT4var19-IE to either primary HBMEC monolayers or engineered 3D brain microvessels was not inhibited by a pool of malaria-naive human unfiltered serum (see Fig. S4 in the supplemental material). To investigate whether IE were simply trapped in 3D microvessels due to altered deformability characteristics, we perfused 2G2, a knobless parasite variant derived from the IT4 parasite genotype, which predominantly expresses *It4var20* (DC8-EPCR-binding PfEMP1 [10]) (Fig. S1). Notably, the knob protrusions display PfEMP1 proteins on the erythrocyte surface and enhance IE binding (41). Unlike IT4var19 parasites, 2G2-IE had negligible binding at regions of WSS greater than 1 dyn/cm^2^ and presented significantly lower binding levels at lower WSS (Fig. 2B). Together, these findings indicate that IE present a characteristic knob and PfEMP1-dependent binding to engineered 3D brain microvessels and there is minimal mechanical trapping of P. falciparum-IE.

10.1128/mBio.00420-19.5 FIG S4 IT4var19 binding in the presence of human serum. IT4var19 binding was compared in binding medium supplemented with 10% FBS or 10% human serum (HS) under (A) static conditions in primary HBMEC monolayers and (B) flow conditions in 3D brain microvessels. (A) Box plot with horizontal line indicating medians and whiskers representing the 10th and 90th percentiles (*n* = 3 biological replicates). ns, not significant. (B) IT4var19 binding in columns 1 and 13 of the 3D microvessels. The median binding levels are represented by dots and interquartile range by colored regions. Statistical analysis of binned regions was determined by a Mann-Whitney nonparametric test (*n* = 4 independent biological replicates). Download FIG S4, PDF file, 0.3 MB. Copyright © 2019 Bernabeu et al. 2019 Bernabeu et al. This content is distributed under the terms of the Creative Commons Attribution 4.0 International license.

10.1128/mBio.00420-19.10 MOVIE S3 Cropped field from Movie S1 showing the distinctive P. falciparum clustered binding pattern in a low-velocity–low-WSS (0.15 dyn/cm^2^) region. The 2-min video was taken at 36 frames/s (fps) and is displayed at 360 fps. Download Movie S3, AVI file, 6.9 MB. Copyright © 2019 Bernabeu et al. 2019 Bernabeu et al. This content is distributed under the terms of the Creative Commons Attribution 4.0 International license.

### P. falciparum*-*IE demonstrate heterogenous binding in resting 3D brain microvessels.

**FIG 3:**
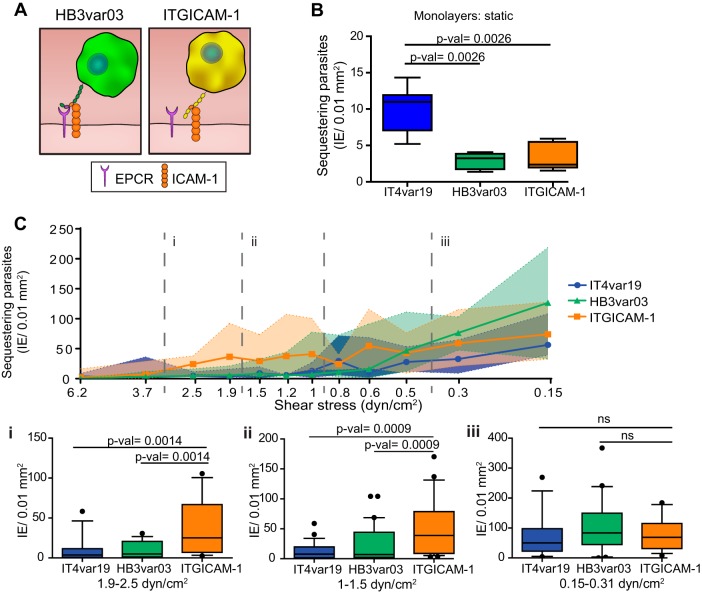
PfEMP1 presents heterogeneous binding in 3D microvessels. (A) Cartoon schematic representation of P. falciparum parasite lines, their respective PfEMP1, and binding properties to endothelial receptors. (B) Binding of IT4var19, HB3var03, and ITGICAM-1 IE to resting HBMEC monolayers in static binding assays. In the box plot, horizontal lines indicate medians, and whiskers show the 10th and 90th percentiles. Statistical significance was determined by a Kruskal-Wallis test corrected for multiple comparisons by the Benjamini, Krieger, and Yekutieli method (*n* = 5 to 9 biological replicates). (C) IT4var19, HB3var03, and ITGICAM-1 IE binding levels at the different estimated WSS regions in 3D microvessels. Medians are represented by dots and interquartile range by colored regions. Statistical analysis of binned regions (dotted lines) is shown in box plots (i, 1.9 to 2.5 dyn/cm^2^; ii, 1 to 1.5 dyn/cm^2^; iii, 0.15 to 0.3 dyn/cm^2^) and was determined by a Kruskal-Wallis test corrected for multiple comparisons by the Benjamini, Krieger, and Yekutieli method (*n* = 4 to 5 independent biological replicates). Horizontal lines indicate medians, whiskers represent the 10th and 90th percentiles, and dots represent minimum and maximum values. ns, not significant.

To further examine the role of PfEMP1 phenotypic diversity in parasite binding, we investigated two ICAM-1 binding parasite lines. ITGICAM-1 predominantly expresses a dual CD36 and ICAM-1 binder from group B PfEMP1 (*It4var16*) (13, 15), and HB3var03 predominantly expresses a dual EPCR and ICAM-1 binder from group A PfEMP1 (*Hb3var03*) (13, 14, 25) (Fig. 3A; Fig. S2). Based on binding to transfected CHO-ICAM-1 cell lines and recombinant ICAM-1, ITGICAM-1 is considered to have a higher ICAM-1 binding affinity than HB3var03 (13). As previously shown under static binding conditions (13, 36), HB3var03 and ITGICAM-1 have significantly lower levels of cytoadhesion to HBMEC monolayers than IT4var19 (Fig. 3B). However, when perfused on 3D brain microvessels, binding levels were increased by 25- and 30-fold, respectively, at low WSS (<0.31 dyn/cm^2^) (compare Fig. 3B and C), and both ICAM-1 binding variants had median binding levels similar to or higher than those of IT4var19 (DC8-EPCR) across all WSS in 3D microvessels (Fig. 3C). In addition, ITGICAM-1, presented significantly higher binding at regions of medium and high WSS (1.0 to 2.5 dyn/cm^2^) than both IT4var19 (EPCR) and HB3var03 (dual EPCR and ICAM-1) (Fig. 3C), consistent with a major role of ICAM-1 in mediating P. falciparum adhesion under flow (42, 43). As our model mimics the dynamic fluidic properties of the arteriole-capillary-venule unit, the diverse parasite binding at higher WSS might provide an adaptation to bind to different vessel beds or dimensions (44).

### P. falciparum-IE expressing different PfEMP1 respond differently to activated 3D microvessels.

P. falciparum-IE are likely to encounter different levels of endothelium activation as infected individuals progress to severe disease, in part because of increased systemic levels of tumor necrosis factor alpha (TNF-α) (26, 45). Significantly, TNF-α has opposing effects on EPCR and ICAM-1 surface expression levels on endothelial cells, but currently little is known about how endothelial activation status influences the affinity of parasite variants that typify severe malaria infections (18, 19, 22, 23, 46). Although recent studies have addressed how group A EPCR-binding parasites interact with resting and activated endothelium (13, 14), neither study examined DC8-EPCR variants.

**FIG 4:**
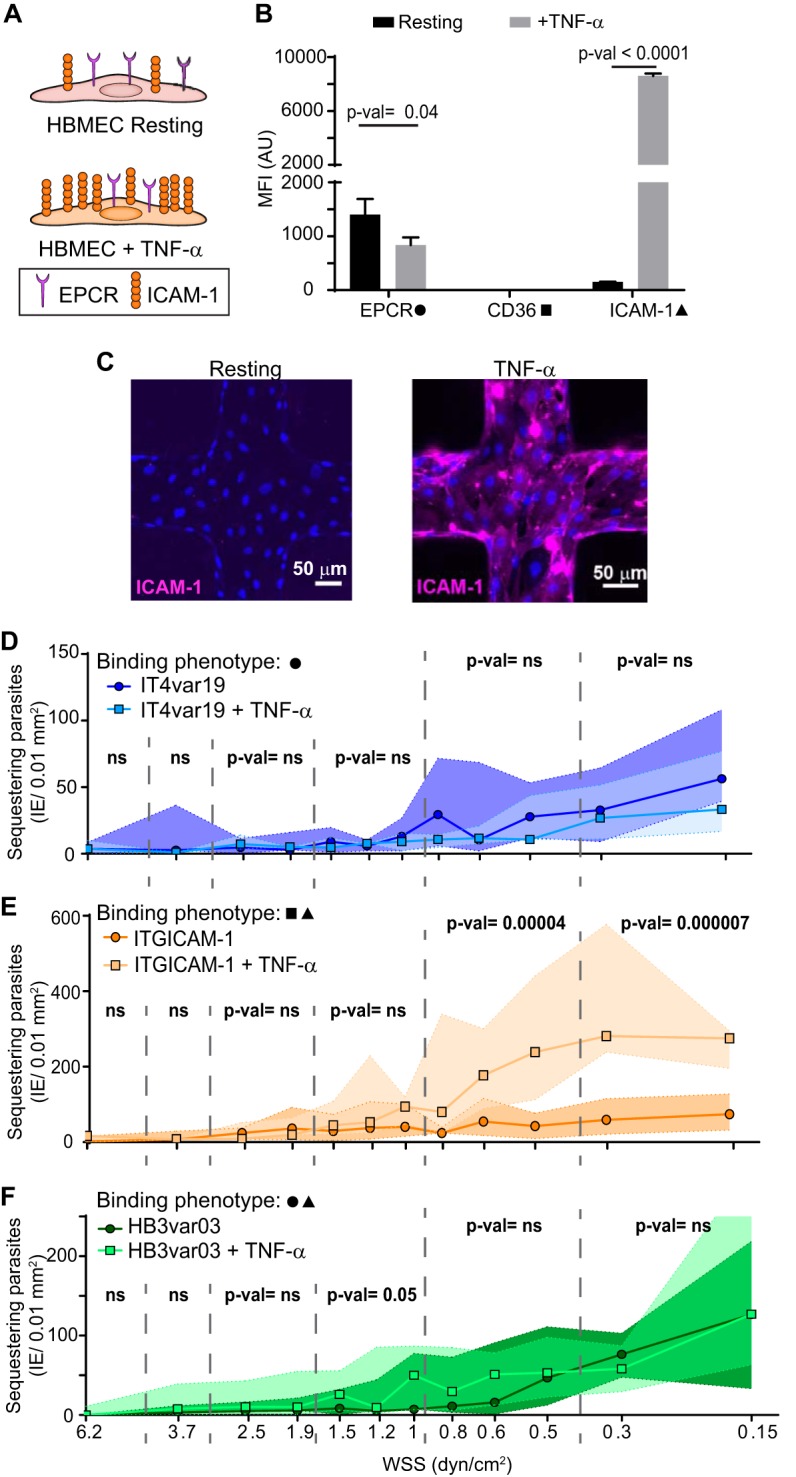
P. falciparum binding to resting and TNF-α-stimulated 3D brain microvessels. (A) Schematic representation of surface expression of receptors on primary HBMEC under resting and activated conditions. (B) Surface receptor expression measured by flow cytometry on resting HBMEC monolayers and after 18 h of stimulation with TNF-α. Bars represent mean ± standard deviation (SD) (*n* = 3 biological replicates). Statistical analysis was determined by an unpaired *t* test. MFI (AU), mean fluorescence intensity (arbitrary units). (C) IFA z-projection of resting and activated 3D brain microvessels stained with an anti-ICAM-1 antibody (magenta). (D to F) Binding levels of IE to resting and TNF-α-activated (18 h) 3D microvessels. Dots represent median binding levels, and the colored area represents the interquartile range (*n* = 4 to 5 independent biological replicates). Statistical analysis of binned regions was determined by Mann-Whitney test.

An 18-h treatment of primary HBMEC with TNF-α led to an average 40% decrease of EPCR and a 50-fold increase of ICAM-1 expression (Fig. 4A to C; see Fig. S5 in the supplemental material). Notably, the three parasite lines displayed different, flow-rate-dependent binding phenotypes on resting and TNF-α-stimulated 3D brain microvessels. Whereas TNF-α led to a nonsignificant binding reduction of IT4var19-IE (DC8-EPCR binder) across all WSS, binding of HB3var03-IE (dual EPCR and ICAM-1) presented a moderate and significant increase in binding at regions between 1 and 1.5 dyn/cm^2^, and ITGICAM-1-IE (dual CD36 and ICAM-1) presented a significant increase in binding at WSS regions <1 dyn/cm^2^ (Fig. 4D to F).

10.1128/mBio.00420-19.6 FIG S5 HBMEC surface expression of endothelial receptors. A representative histogram shows levels of endothelial receptor surface expression on resting or activated HBMECs after 18 h of stimulation with TNF-α. EPCR and CD36 were stained with Alexa Fluor 488- and fluorescein isothiocyanate (FITC)-conjugated antibodies, respectively. ICAM-1 and CD31 were stained with phycoerythrin (PE)-conjugated antibodies. An IgG control was used as a negative control. The average surface expression levels are shown in Fig. 4B. Download FIG S5, PDF file, 0.4 MB. Copyright © 2019 Bernabeu et al. 2019 Bernabeu et al. This content is distributed under the terms of the Creative Commons Attribution 4.0 International license.

**FIG 5:**
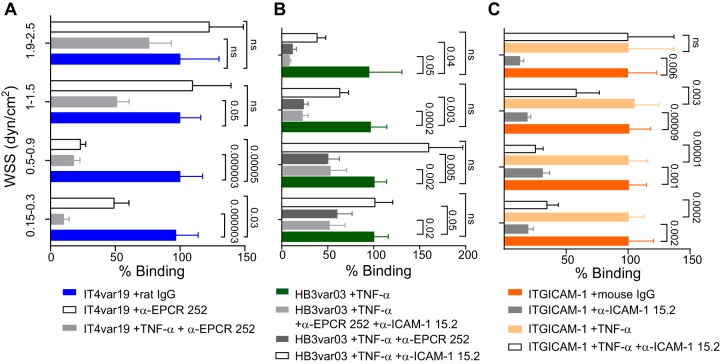
Role of EPCR and ICAM-1 in P. falciparum-IE binding to 3D brain microvessels. (A to C) Percentage of binding of P. falciparum-IE in the presence of anti-EPCR MAb 252 or anti-ICAM-1 MAb 15.2 antibody. Percentage of binding is normalized to binding in the presence of IgG control antibody or the absence of antibody treatment. Bars represent mean ± standard error of the mean (SEM) (*n* = 4 to 5 independent biological replicates). Statistical analysis of binned regions in pairwise comparisons was determined by Mann-Whitney test and in multiple comparisons was corrected by a Kruskal-Wallis test.

To investigate whether differences in the expressed PfEMP1 variant contribute to the various parasite interactions with resting and activated 3D microvessels, we preincubated engineered 3D brain microvessels with inhibitory monoclonal antibodies (MAbs) against EPCR and ICAM-1 before parasite perfusion (Fig. 5). On resting 3D brain microvessels, EPCR blockade significantly decreased the binding of IT4var19 (EPCR binder) at WSS lower than 1 dyn/cm^2^ (Fig. 5A). This inhibition was even greater on TNF-α-activated vessels that have lower EPCR surface expression levels (Fig. 5A). Likewise, EPCR blockade significantly inhibited binding of HB3var03 (dual EPCR and ICAM-1 binder) on activated 3D microvessels at all WSS and was more effective at higher WSS (Fig. 5B). Thus, both parasite lines exhibited sensitivity to EPCR blockade.

Inhibition assays also suggested that ICAM-1 upregulation contributes to the increased parasite binding in activated 3D brain microvessels. For instance, the increased binding of the higher-ICAM-1-affinity parasite line ITGICAM-1 (dual CD36 and ICAM-1) to activated microvessels was abolished by ICAM-1 blockade of 3D microvessels (Fig. 5C). By comparison, ICAM-1 blockade on its own led to a non-statistically significant binding reduction of the lower-ICAM-1-affinity binder HB3var03 to activated vessels. Notably, this reduction only occurred in the regions that presented a binding upregulation after endothelial activation (WSS of >1 dyn/cm^2^) (Fig. 5C). However, anti-EPCR blockade alone or the combination of anti-EPCR and anti-ICAM-1 antibodies (Fig. 5C) significantly decreased HB3var03 binding at higher WSS, suggesting that both receptors cooperated in mediating the enhanced parasite binding. Collectively, these parasite comparisons provide evidence that both EPCR and ICAM-1 contribute to the parasite-brain vessel interaction and that parasites are sensitive to differences in receptor levels following endothelial activation.

### Cytoadhesion of a cerebral malaria isolate in 3D brain microvessels.

**FIG 6:**
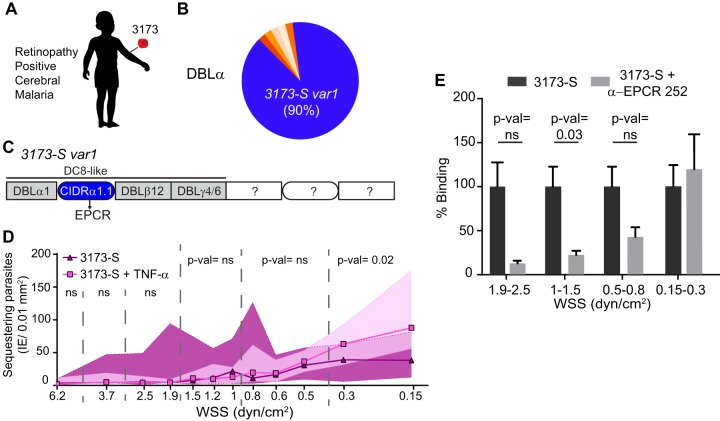
Binding of a cerebral malaria isolate in 3D brain microvessels. (A) 3D microvessels were perfused by a parasite isolate (3173-S) that was cloned by limited dilution from a retinopathy-positive, cerebral malaria patient. (B) Sequencing of N-terminal DBLα tags showed that a single *var* gene accounts for 90% of the total amplified *var* transcripts. (C) Quantitative real-time PCR (qRT-PCR) analysis with *var* domain primers and extension of the dominant DBLα tag sequence into the flanking domains (Fig. S6) revealed that the 3173-S *var1* transcript presents a DC8-like architecture predicted to bind to EPCR. (D) Binding of 3173-S (*n* = 5 to 6 independent biological replicates) to resting and TNF-α-activated (18 h) 3D microvessels. Dots represent median binding levels, and the colored area represents the interquartile range. (E) Percentage of binding of 3173-S in the absence or presence of anti-EPCR MAb 252 inhibitory antibody. The percentage of binding is normalized to the binding levels in the absence of treatment. Bars represent mean ± SEM (*n* = 5 to 6 independent biological replicates). Statistical analysis of binned regions in panels D and E was determined by Mann-Whitney test.

To better understand the linkage between cytoadhesion phenotypes and disease pathogenesis, we perfused a parasite line that was isolated and cloned by limiting dilution from a retinopathy-positive, pediatric CM patient (Fig. 6A). Notably, retinal pathology is strongly linked to histologically confirmed pediatric CM (47). Amplification of *var* sequence “tags” using primers that target semiconserved regions in the N-terminal DBLα domain revealed that the 3173 clonal line, renamed 3173-S for this study, expressed a single predominant *var* transcript (*3173-S var1* = 90% of the total amplified DBLα tags). This *var* gene encoded a group A DBLα domain followed by a DC8-like cassette predicted to bind EPCR (Fig. 6B and C; see Fig. S6 in the supplemental material). The binding behavior of 3173-S-IE was similar to that of IT4var19-IE, in that both DC8 *var*-expressing parasite lines displayed lower binding activity to TNF-α-stimulated 3D brain microvessels, especially at WSS higher than 0.5 dyn/cm^2^ (compare Fig. 4D and 6D). Furthermore, anti-EPCR antibodies significantly reduced 3173-S binding to resting 3D microvessels at higher WSS (Fig. 6E). Taken together, this suggests that EPCR contributes to 3173-S binding and that DC8-parasite cytoadhesion is sensitive to loss of EPCR expression levels on activated 3D microvessels. Conversely, at the lowest-WSS regions (<0.5 dyn/cm^2^), 3173-S binding was slightly increased after endothelial activation and EPCR blockade, suggesting the potential involvement of other coreceptors in DC8-PfEMP1 binding at lower WSS.

10.1128/mBio.00420-19.7 FIG S6 3173-S cerebral malaria isolate characterization. (A) A qRT-PCR strategy used to characterize the *var* transcriptional profile of the P. falciparum 3173-S isolate cloned by limiting dilution from a retinopathy-positive, cerebral malaria patient. (i) Schematic representation of the DC8-like cassette and primers that target it. (ii) Transcription of different *var* domains was analyzed by the primers of Lavstsen et al. (black) (18), Mkumbaye et al. (blue)(20), and Lennartz et al. (red) (14). Transcription unit levels are normalized to the housekeeping control gene coding for STS (seryl-tRNA synthetase). (B) Schematic representation of the sequencing strategy followed to amplify the DC8-like domain of strain 3173-S. Primers targeting semiconserved regions in the N-terminal DBL domain of most *var* genes were used to amplify DBLα tags from 3173-S. This approach was used to estimate the number and proportion of different *var* transcripts being transcribed. The most common DBLα tag (designated 3173-S *var1*) was extended to the flanking DBLβ12 domain in the DC8-like cassette. (C) A neighbor-joining tree of 45 previously annotated CIDRα1 sequences (58) showed that 3173-S *var1* contained a CIDRα1.1 domain, which is characteristic of the DC8 cassette. Download FIG S6, PDF file, 0.5 MB. Copyright © 2019 Bernabeu et al. 2019 Bernabeu et al. This content is distributed under the terms of the Creative Commons Attribution 4.0 International license.

## DISCUSSION

Although it has been recognized since the 1880s that cerebral malaria is associated with sequestration of P. falciparum-IE on the cerebral microvascular walls, large knowledge gaps remain in cerebral malaria pathogenesis. Multiple studies have revealed that parasites expressing EPCR binding PfEMP1 (EPCR only and dual EPCR-ICAM-1) are enriched in the peripheral blood of CM patients (14, 19, 22). However, there is still debate about the specific PfEMP1-host receptor interactions involved in cerebral binding.

Here, we introduce a novel flow-based 3D human brain microvessel model that reproduces the characteristic knob-dependent sequestration of P. falciparum-IE on the endothelial lining and is suitable for modeling small arterioles or postcapillary venules where P. falciparum-IE are known to sequester (44). The main limitation of our model is the low throughput, as 3D brain microvessel fabrication is complex and requires multistep skills, including microfabrication, molding, and device assembly. Additionally, the use of a soft collagen matrix may have caused small variations in vessel diameter between 3D microvessel devices, which may affect the local flow characteristics and contribute to parasite binding variability across experiments. Nevertheless, it confers the advantage of simultaneous study of P. falciparum sequestration at a wide range of physiological WSS and flow velocities within a single device. Although P. falciparum IE were perfused at an unphysiologically low hematocrit to avoid vessel clogging, parasites had higher binding levels in 3D microvessels than conventional static binding assays in cell monolayers. Future studies could explore the use of physiological hematocrits, as uninfected erythrocytes might increase the margination of IE (48) and increase sequestration rates within our device.

**FIG 7:**
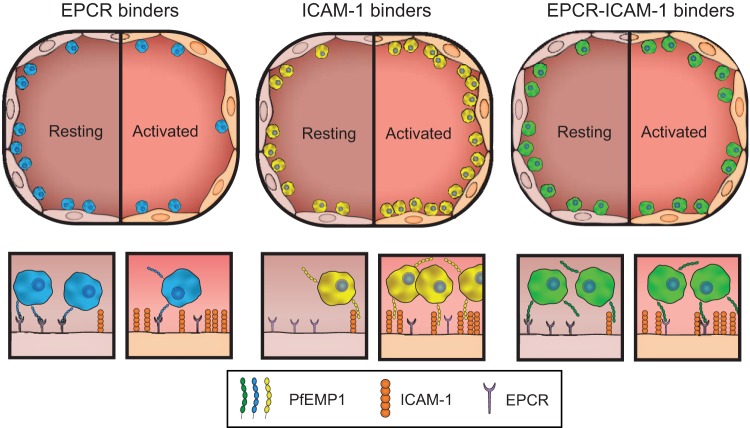
P. falciparum presents heterogenous binding to resting and activated 3D brain microvessels. Hypothesized model of parasites with different cytoadhesion phenotypes binding at different stages of infection: asymptomatic/early infection (resting endothelium) and late infection with disease (activated endothelium). Overall, the three parasite lines display similar binding levels on resting endothelium, except ITGICAM-1 had higher binding levels at WSS of >1 dyn/cm^2^. Conversely, the parasite lines diverge in response to endothelium activation. IT4var19 and 3173-S (DC8-EPCR binders) presented a reduction in binding to activated endothelium, ITGICAM-1 (dual CD36 and high ICAM-1 affinity) presented an increase of binding to activated endothelium, and HB3var03 (dual EPCR and low ICAM-1 affinity) presents similar binding levels in both states. The lower schematic illustrates how PfEMP1 variants with different combinatorial binding properties may provide the parasite with a sophisticated strategy to rapidly adapt to changes in EPCR and ICAM-1 expression levels that accompany endothelial activation.

P. falciparum infection is characterized by a systemic endothelial activation and colocalization of IE on ICAM-1-positive cerebral vasculature (26). It has been hypothesized that this would select for parasite binding variants with higher binding affinity for activated endothelium, but there has been limited investigation of this concept. Our central finding here is that PfEMP1 variants linked to severe malaria display extensive heterogeneity in binding activity for resting and activated 3D brain microvessels, and differences are at least partially attributable to their combinatorial binding properties for EPCR and ICAM-1. Although EPCR binding appears to be an ancient parasite adhesion trait that has been retained within the PfEMP1 family across the large time span that separates human and chimpanzee malaria species (9, 49), and several studies have converged toward its importance for parasite cytoadhesion (10, 13, 14, 26, 35, 36), it recently has been questioned whether EPCR is a physiological endothelial receptor for P. falciparum (40). Assays on engineered 3D brain microvessel provide evidence that EPCR and ICAM-1 contribute to parasite binding heterogeneity (as modeled in Fig. 7). Of interest, the only parasite line in this study that presented increased binding to activated endothelium was ITGICAM-1, as expected by its strong affinity to ICAM-1. However, this PfEMP1 variant has dual CD36 and ICAM-1 binding properties that may lead to preferential sequestration in vascular beads with high CD36 expression, which is absent in the brain. Conversely, the dual EPCR-ICAM-1 binder HB3var03 parasite line displayed a versatile binding phenotype with similar binding levels in both resting and activated vessels. Finally, binding of two parasites lines expressing DC8-PfEMP1 (IT4var19 and 3173-S: EPCR binders) was slightly reduced to TNF-α-stimulated endothelial cells. These findings highlight the considerable phenotypic heterogeneity between parasite lines expressing different PfEMP1 variants and illuminate how PfEMP1 combinatorial binding properties may influence parasite affinity for resting and activated endothelial cells.

This study has important implications for severe malaria vaccine design. Our results show that anti-EPCR strategies significantly inhibit the binding of both DC8-PfEMP1 (IT4var19 and 3173-S) and dual EPCR and ICAM-1 binding (HB3var03) parasite lines to 3D brain microvessels, especially at physiological WSS of >0.5 dyn/cm^2^. Although ICAM-1 blockade alone was insufficient to prevent the increased binding of a dual EPCR and ICAM-1 binding parasite line to activated 3D brain microvessels, our findings show a cooperative role of both receptors in parasite adhesion at higher WSS. Altogether, these studies suggest that an antiadhesive strategy focused on targeting both EPCR and ICAM-1 may be most effective.

EPCR plays a key role in maintaining vascular homeostasis, as it exerts protective pathways through activation of protein C. By binding to EPCR and impairing its cytoprotective and anticoagulant functions (21, 22, 35, 36, 50), cytoadherent IE may act as a force multiplier to amplify other prothrombotic and barrier disruptive mechanisms in cerebral microvessels (51). To better understand blood-brain barrier alterations on CM patients, future engineered 3D microvessel approaches toward a more physiological model could incorporate perivascular cells, such as pericytes and astrocytes in the collagen matrix, and add other blood components to the lumen to investigate the role that human factors play in CM complications. Additionally, as CM patients present a widespread sequestration in multiple vascular beds (17, 23, 52), future studies can take advantage of the versatility of our model (28, 34, 53–55) to study the avidity and consequences of IE binding to other vascular sites. In conclusion, our study reveals heterogeneity in parasite binding to resting and activated endothelial cells using a new flow-based 3D brain microvessel model *in vitro* platform that can be used to investigate host cell or pathogen interactions with brain endothelium.

## MATERIALS AND METHODS

### Parasite lines.

P. falciparum parasites were cultured using human O+ erythrocytes (Valley Biomedical) in RPMI 1640 medium (GIBCO) supplemented with 10% human type A-positive serum (for lab-adapted parasite lines) or 5% Albumax (for the adapted CM isolate). Parasite lines were grown in a gas mixture of 90% N_2_, 5% CO_2_, and 5% O_2_, except for the recently adapted CM isolate, which was grown in a gas mixture of 94% N_2_, 5% CO_2_, and 1% O_2_. The ITGICAM-1 (56), IT4var19 (24), and 2G2 (15) parasite lines are isogenically related. The field isolate 3173 was recovered from the peripheral blood of a retinopathy-positive, cerebral malaria patient. This parasite was subjected to limited dilution cloning and renamed 3173-S after growth and expansion in Seattle.

### Human subject enrollment criteria and ethics statement.

During the 2013 malaria season, the pediatric cerebral malaria retinopathy-positive patient from whom the 3173 parasite was isolated was recruited to the study at Queen Elizabeth Central Hospital (QECH) in Blantyre, Malawi. At admission, the patient had a Blantyre coma score of 2. His parents consented to his enrollment in the study, and 2 ml of blood was drawn into citrate anticoagulant. Sample collection was approved by the institutional review boards at the University of Malawi College of Medicine and Michigan State University. Before patient enrollment, the parent/guardian of the study participant gave written informed consent.

### Human brain microvascular endothelial cell culture.

Primary HBMEC (Cell Systems; ACBRI 376) were cultured and expanded according to the manufacturer’s recommendations in CSC complete medium (Cell Systems; 4Z0-500) containing 10% fetal calf serum (FCS). Cells were grown as a monolayer with attachment factor as growth support (4Z0-210; Cell Systems) until seeded in microvessels up to passage 8.

### 3D brain microvessel fabrication.

Type I collagen was isolated from rat tails (Rockland; RT-T297), lyophilized, and resuspended to a stock concentration of 15 mg/ml in 0.1% acetic acid and then diluted and neutralized to 7.5 mg/ml on ice before microvessel fabrication. Microvessels were fabricated using soft lithography and injection molding as previously described (28). Briefly, the top part of the microvessels is composed of a negative impression of the network and generated by injecting collagen into the space created between the top plexiglass jig and an oxygen plasma-treated polydimethylsiloxane (PDMS) stamp. Inlet and outlet ports of the microvessel network were defined by inserting stainless steel dowel pins in two holes of the top jig before injecting the collagen. The bottom part of the microvessels is composed of a flat layer of collagen, compression molded by a flat PDMS stamp on top of a bottom jig covered with a 22- by 22-mm coverslip. After 20 min of gelation at 37°C, the PDMS stamps were removed, and the top jig containing an open microfluidic network was mechanically sealed against the bottom jig containing a flat collagen layer. The device was then incubated with CSC complete medium for 1 h followed by HBMEC seeding. Primary HBMEC at a concentration of 7 × 10^6^ cells/ml (10 μl in volume) were seeded from the inlet and distributed into the microfluidic network by gravity-driven flow. After 1 h of incubation at 37°C, additional medium was introduced into the network to remove excessive cells that were not attached. Microvessels were fed every 12 h by gravity-driven flow, where medium was added to the inlet port and removed from the outlet port to create a height difference between media of around 8.5 mm and a corresponding pressure drop of around 80 Pa. This initial height and pressure difference equilibrated over approximately 2 h, defining a unidirectional, decaying flow across the microvessel network with a maximum shear stress of 6.1 dyn/cm^2^ and a time-averaged shear stress of 0.1 dyn/cm^2^. Microvessels were cultured for 3 to 5 days, and based on a visual quality inspection, ∼50% of individual devices were selected for parasite perfusion studies. Devices with bubbles or visible imperfections in the microvascular lumen were excluded from parasite binding assays but were utilized for antibody labeling.

### WSS numerical simulation.

The flow characteristics of 3D brain microvessels during IE perfusion were simulated using COMSOL Multiphysics software (package version 5.1). Because IE perfusion occurred at relatively slow flow velocity in small-diameter microvessels, flow in the microvessel network was assumed to be laminar, and the stationary solver for laminar flow was used with predefined Navier-Stokes equations. Due to the low hematocrit (∼0.04%) used during perfusion, flow was assumed to be Newtonian, and WSS was calculated based on fluid viscosity at 1 cP, similar to water or culture medium at room temperature (viscosity of 10^−3 ^Pa s and density of 10^3^ kg/m^3^). The inlet boundary conditions were defined for the perfusion flow rate of 10 μl/min, and the outlet boundary conditions were set at zero pressure. Flow velocity, pressure, and shear stress from the converged solution were imported into MATLAB 2017a via LiveLink.

### Parasite binding.

Mature-stage P. falciparum-IE were enriched by a MACS cell separator (LD columns, Miltenyi Biotec), and an enriched 60 to 80% IE suspension was diluted to 5 × 10^6^ IE/ml in binding medium. Serum-containing CSC complete medium (10% fetal bovine serum) was used as a binding medium in most experiments, except for experiments to test serum inhibition of parasite binding (Fig. S4), in which binding medium was prepared with EBM-2 supplemented with an EGM-2 MV Bulletkit (Lonza; cc-3202) and with either 10% fetal bovine serum or 10% pooled human unfiltered serum.

### Static-monolayer binding assays.

Primary HBMECs were seeded onto collagen-coated eight-well slides (BD BioCoat; Falcon) and grown for 2 to 3 days until reaching confluence. Three hundred microliters of IE (5 × 10^6^ IE/ml) suspension in prewarmed binding medium was incubated in each well at 37°C for 30 min. Nonbound IE were washed by flipping the slides in a gravity wash for 10 min. The slides were fixed in 3.7% paraformaldehyde (PFA) for 20 min, and nuclei were stained with 4′,6-diamidino-2-phenylindole (DAPI [8 μg/ml]) for 1 h. Human serum inhibition experiments were done at a concentration of 2.5 × 10^6^ IE/ml.

### Controlled flow binding assays in 3D brain microvessels.

3D brain microvessels were grown for 3 to 5 days. To avoid clogging of the 3D microvessel devices, P. falciparum*-*IE were perfused at a low concentration (5 × 10^6^ IE/ml) for 30 min at a flow rate of 10 μl/min using a syringe infusion pump (KD Scientific Inc.; KDS220) at room temperature, followed by a 10-min wash with binding medium at the same flow rate. HBMEC microvessels were fixed in 3.7% PFA for 20 min and then permeabilized with 0.1% Triton X-100 in phosphate-buffered saline (PBS) and stained with DAPI (8 μg/ml) for 1 h. Binding experiments on activated 3D microvessels were performed after an 18-h stimulation with TNF-α (10 ng/ml) at 37°C. For IE binding inhibition experiments, microvessels were pretreated for 20 min before IE perfusion by gravity-driven flow with mouse anti-human ICAM-1 monoclonal antibody (MAb) 15.2 (Abcam; ab20 [5 μg/ml]), rat anti-human EPCR MAb 252 (Sigma-Aldrich; E6280-200UL [50 μg/ml]), or mouse (Abcam; ab19443) or rat (Thermo Fisher; 14-4301-85) IgG isotype control. Human serum inhibition experiments were done at a concentration of 2.5 × 10^6^ IE/ml, and videos were done at a concentration of 2 × 10^7^ IE/ml. For each experimental condition, a 3D microvessel device was used once.

### Parasite binding quantification.

Binding was determined by counting the number of parasite DAPI-stained nuclei in images taken at ×200 magnification in a Nikon TiE inverted wide-field microscope. For static binding assays, bound IE were quantified in 10 random fields per well. For 3D microvessels, each device presents a 13-by-13 grid with diagonal symmetry, including two regions with identical velocity and shear stress. For instance, the flow velocity decreases along column 1 and increases along column 13 in the same stepwise fashion (Fig. S3). Digital images were acquired for each branch along the outer columns (columns 1 and 13) to avoid the effect of flow interdigitation present in the interior columns of the grid system. Columns 1 and 13 were considered a technical duplicate for each microvessel device (for example, *n* = 4 independent biological replicates means that IE binding was quantified in columns 1 and 13 from four different 3D microvessel devices). Images were acquired at a 3-μm z-step size, and projection images of the bottom of the vessel were produced from z-stacks using Fiji (ImageJ v1.52b) software. The counting strategy is summarized in Fig. S3. To avoid flow artifacts, parasite binding was only determined in network regions where flow was fully developed, unidirectional, and uniform along a large (>0.01-mm^2^) area. Junctions between branches were computationally predicted to display nonuniform shear stresses across their length and were therefore excluded from analysis. Within each branch, three regions of interest along the center wall were averaged to determine parasite binding per area. Entry and exit regions were excluded, as flow is not fully developed in these regions. Parasite binding in regions <10 μm from channel edges was also excluded from the quantification, as unspecific IE trapping might occur in this area due to slight imperfections at regions of collagen sealing or secondary flows (Fig. S3). Observers were blind during image acquisition and parasite counting, as devices are imprinted with a numerical code. This allowed the blind counts to be linked to specific experimental condition during statistical analysis.

### Immunofluorescence microscopy.

All steps in immunofluorescence assays (IFA) were done using gravity flow conditions. 3D brain microvessels were fixed *in situ* in 3.7% PFA and washed twice with PBS, then incubated in Background Buster (Innovex) for 30 min and blocking buffer (2% bovine serum albumin, 0.1% Triton-X in PBS) for 1 h. Blocked microvessels were incubated overnight in primary antibodies diluted in blocking buffer: from Abcam, rabbit anti-caveolin-1, ab2910, 1:100; rabbit anti-VE-cadherin, ab33168, 1:100; fluorescein isothiocyanate (FITC)-conjugated sheep anti-VWF, ab8822, 1:100; mouse anti-ICAM-1, ab2213, 1:50; and mouse anti-GLUT-1, ab40084, 1:50; from Cell Signaling Technology, rabbit anti-β-catenin, 9587, 1:200; and from BD Pharmingen, phycoerythrin (PE)-conjugated mouse anti-CD31 MAb, clone WM59, 1:50. Microvessels were washed three times for 10 min with PBS and incubated with (1:100) secondary antibodies (Thermo Fisher, goat anti-rabbit Alexa Fluor 488, A11034; goat anti-rabbit Alexa Fluor 568, A11036; goat anti-mouse Alexa Fluor 568, A11031; goat anti-rabbit Alexa Fluor 647, A21244; and goat anti-mouse Alexa Fluor 647, A21235) containing DAPI (8 μg/ml) for 1 h. After three 10-min washes with PBS, vessels were imaged using a Nikon A1R confocal microscope, and Image stacks were acquired with an ∼3-μm z-step distance between optical slices. Cross-sections and projections were produced from z-stacks using Fiji (ImageJ v1.52b) or Imaris (v9.1.2) software.

### Statistical analysis.

GraphPad Prism 7 was used for statistical analysis. Pairwise comparisons were analyzed by the nonparametric Mann-Whitney test and three-wise comparisons were analyzed by a nonparametric one-way analysis of variance Kruskal-Wallis test. A *P* value of <0.05 was considered significant. For 3D microvessel binding experiments, quantifications on columns 1 and 13 are considered a technical replicate. To account for small differences in vessel diameter that can alter the estimated shear stress, our statistical analysis of IE binding binned adjacent regions into shear stress increments of ∼0.5 dyn/cm^2^. Due to the limitations of vessel fabrication, the statistical comparisons shown in this article were not done in parallel experiments. Parasite binding conditions with low variability were repeated in 4 independently fabricated 3D microvessel devices, and parasite binding conditions with high variability were repeated in 5 to 6 independent 3D microvessel devices.

10.1128/mBio.00420-19.1 TEXT S1 Supplemental methods. Download Text S1, DOCX file, 0.1 MB. Copyright © 2019 Bernabeu et al. 2019 Bernabeu et al. This content is distributed under the terms of the Creative Commons Attribution 4.0 International license.
