# Supplementary figures and images for: Binding Heterogeneity of Plasmodium falciparum to Engineered 3D Brain Microvessels Is Mediated by EPCR and ICAM-1
Source: mBio. 2019 May 28;10(3):e00420-19. doi: 10.1128/mBio.00420-19 (PMC6538777; doi:10.1128/mBio.00420-19)

**A**

**IT4VAR19 (DC8)**

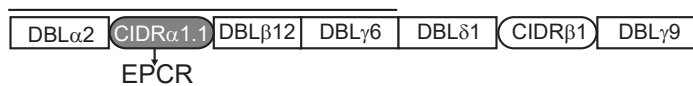

**ITVAR20: 2G2 Knobless (DC8)**

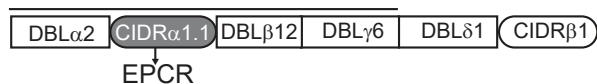

**HB3VAR03 (GroupA)**

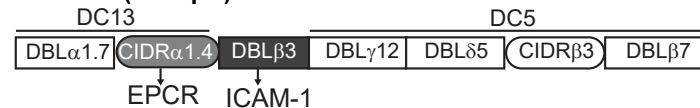

**IT4VAR16: ITGICAM-1 (Group B)**

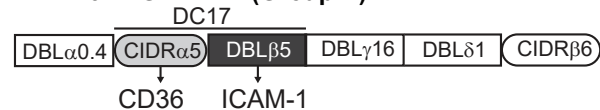

**B**

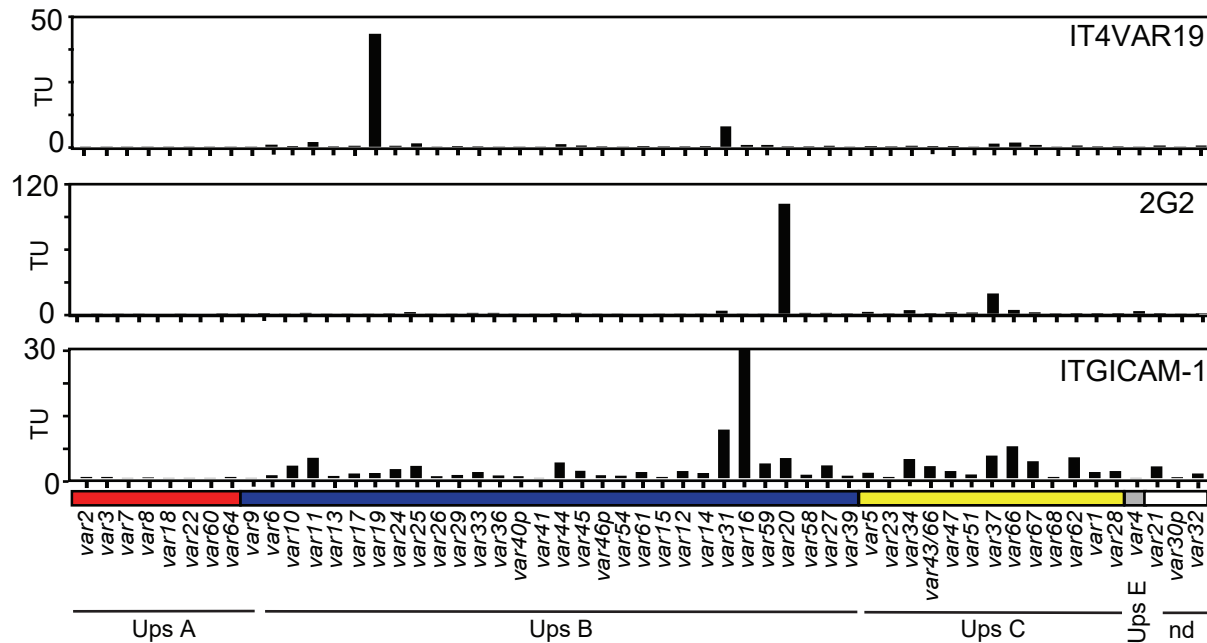

**C**

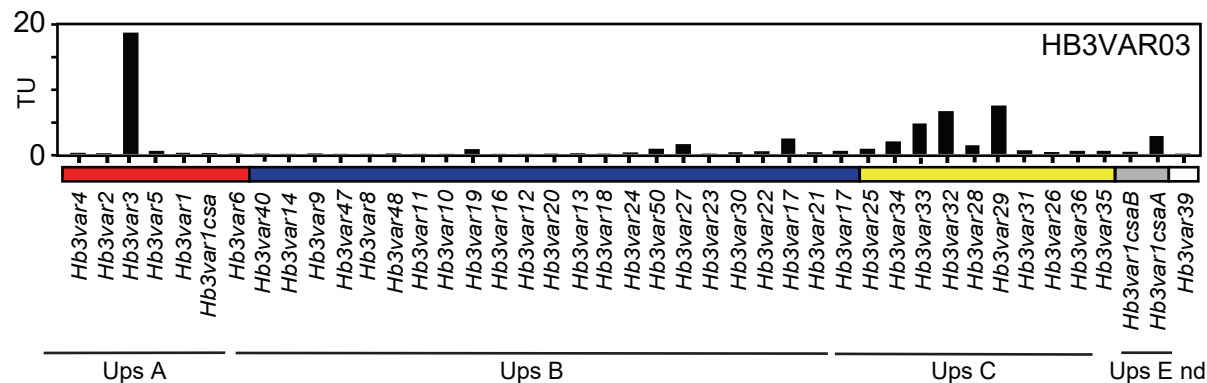

Supplement: FIG S2 [file mBio.00420-19-sf002.pdf]

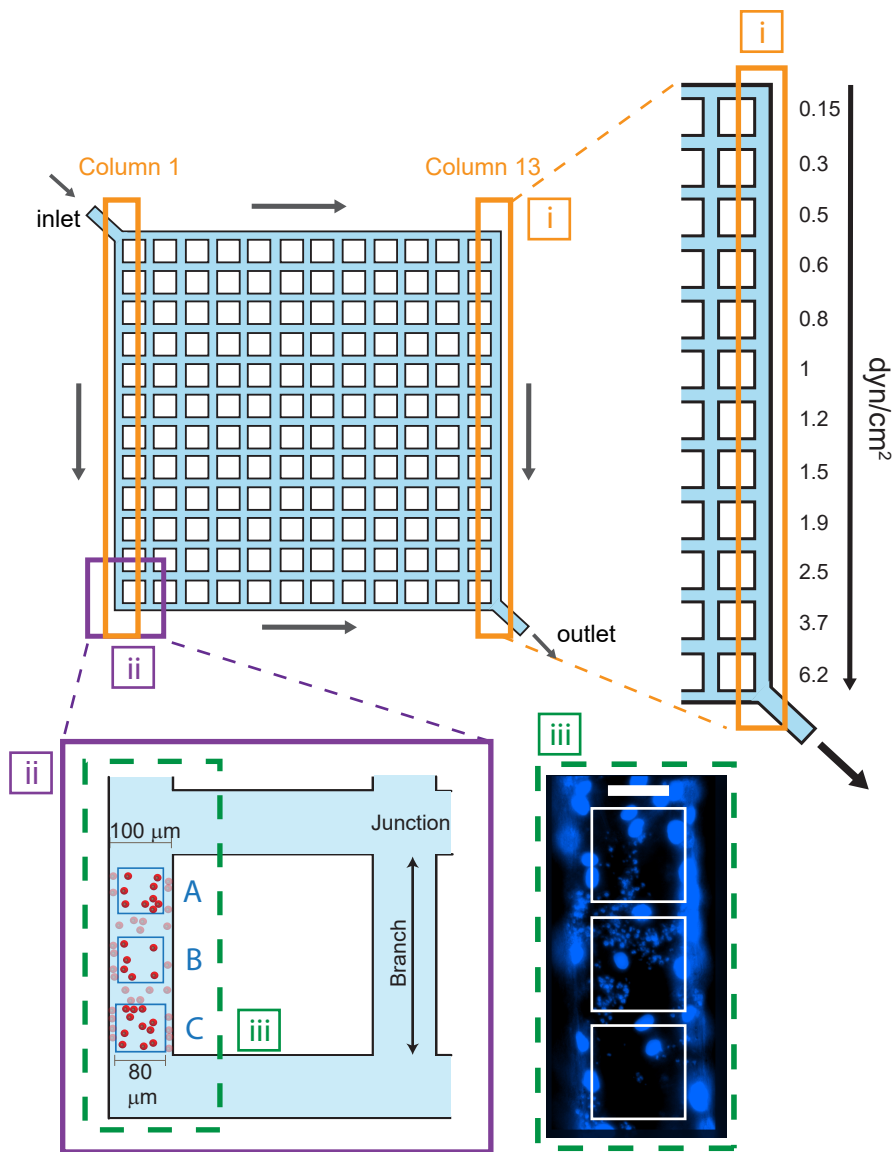

**IE binding**  
Average binding A, B and C

Supplement: FIG S3 [file mBio.00420-19-sf003.pdf]

**A**

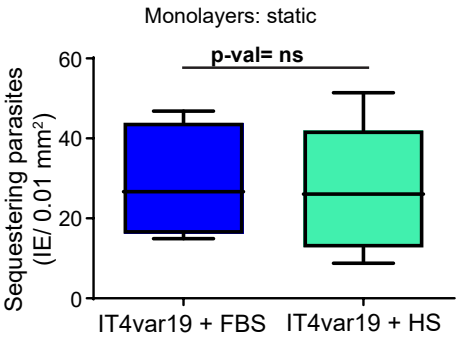

**B**

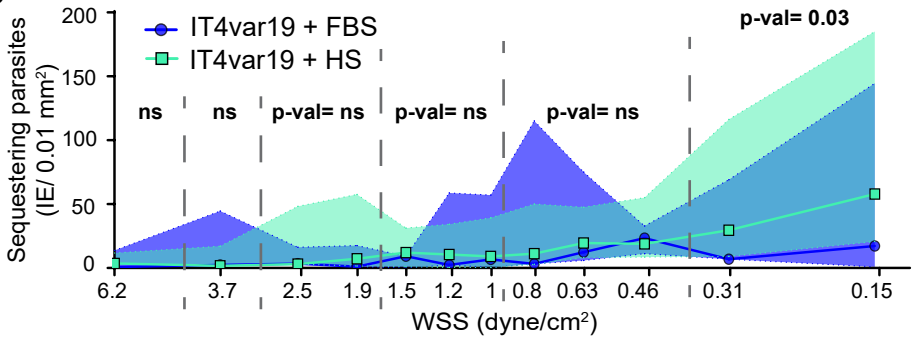

Supplement: FIG S4 [file mBio.00420-19-sf004.pdf]

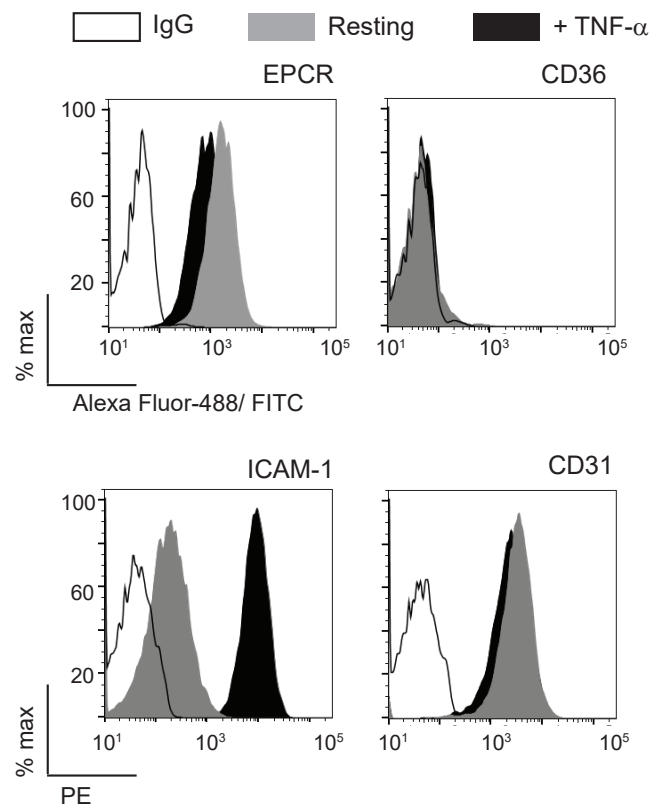

Supplement: FIG S5 [file mBio.00420-19-sf005.pdf]

A

i

Q-RT-PCR strategy

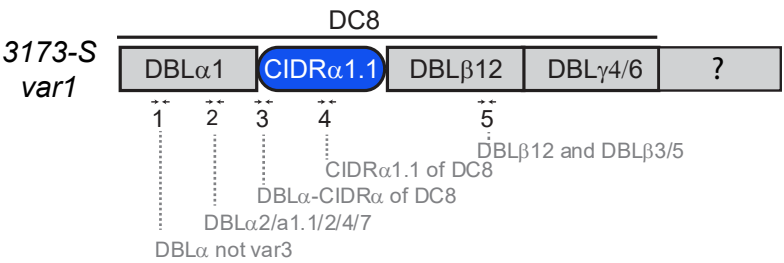

B

Sequencing strategy

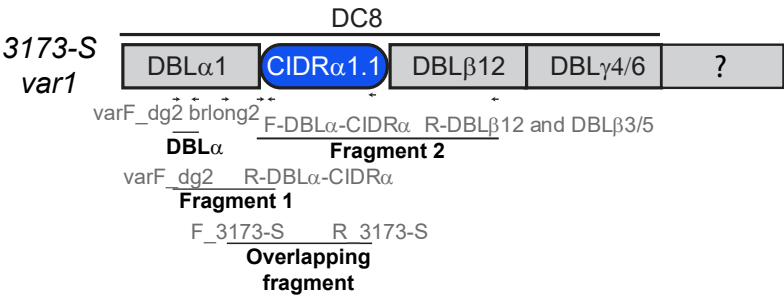

C

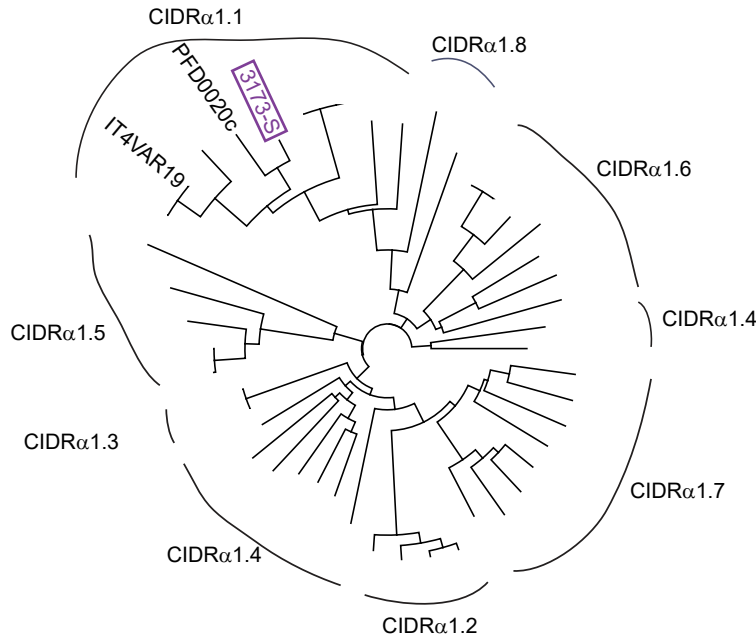

ii

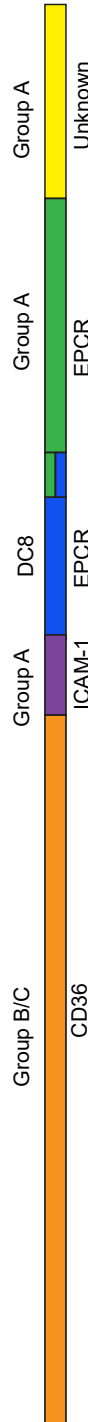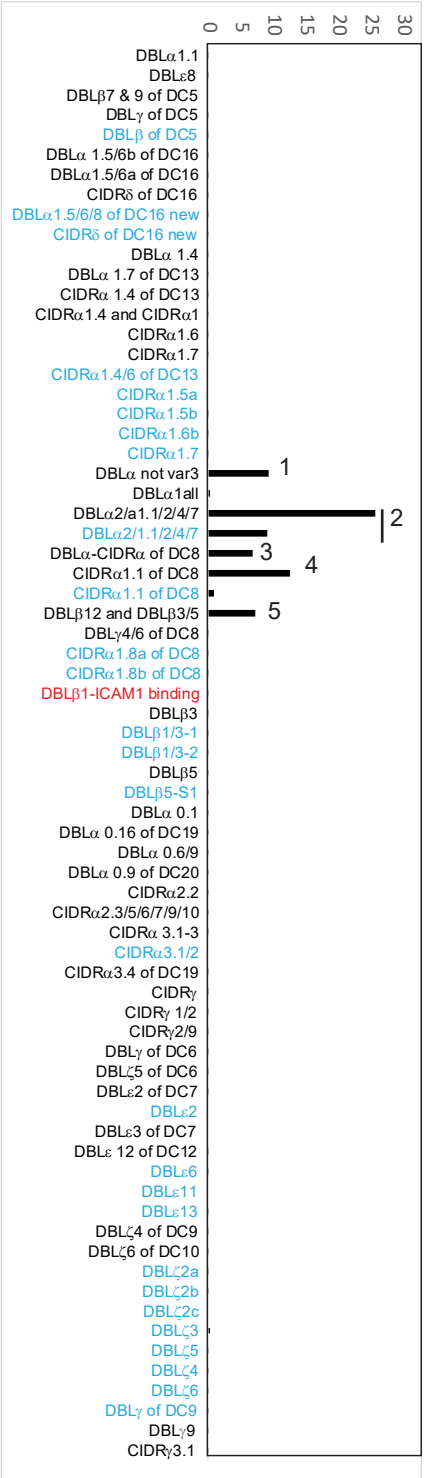

Supplement: FIG S6 [file mBio.00420-19-sf006.pdf]
