# Supplementary material for: Binding Heterogeneity of Plasmodium falciparum to Engineered 3D Brain Microvessels Is Mediated by EPCR and ICAM-1
Source: mBio. 2019 May 28;10(3):e00420-19. doi: 10.1128/mBio.00420-19 (PMC6538777; doi:10.1128/mBio.00420-19)
Supplement: TEXT S1 [file mBio.00420-19-s0001.docx]

**Supplementary methods**

**Determination of *var* transcription by qRT-PCR.** The *var* transcription profile of the parasite lines was regularly monitored by qRT-PCR with a respective set of *var* gene specific primers to the IT4 repertoire (1), the HB3 repertoire (2), or with a set of PfEMP1 domain specific degenerate primers (3-5) for the 3173-S parasite line. Because *var* genes can switch expression, RNA was obtained from synchronized ring parasites (8-12 h post-invasion) in the same time frame as parasite binding assays. RNA was extracted in Trizol LS (Invitrogen) and reversed transcribed using random hexamers and MultiScribe reverse transcriptase (Thermo Fisher). qRT-PCR reactions were performed using Power-SYBR green Master Mix (Thermo Fisher) in an ABI Prism 7500 Thermal Cycler following published amplification conditions for each primer repertoire (1-4). Relative transcription of *var* genes was determined by normalization to the control housekeeping gene seryl-tRNA-synthetase (STS; PF07_0073). The level of expression was represented as transcript units (TU) and calculated as TU = 2^( - ∆CT)^ for the IT4 and HB3 specific primers, and as TU = 2^(5 - ∆CT)^ for domain specific primers (3).

**Characterization of *var* transcription in pediatric cerebral malaria isolate.** The sequencing strategy for the 3173-S parasite line is summarized in Fig. S6. The proportion of *var* genes transcribed was determined by sequencing of DBLα tags using the previously described varF_dg2 and brlong2 primers (3). PCR amplification was carried out using Phusion High-Fidelity DNA polymerase (New England Biolabs) and final primer concentrations of 0.5 mM as follows: 98^o^C for 45 s, followed by 40 cycles of 98^o^C for 10 s, 50^o^C for 20 s, 68^o^C for 20 s and final extension at 72^o^C for 7 min. The amplified fragment was purified and cloned into the PCR-Blunt II- TOPO plasmid. The proportion of expressed *var* transcripts was determined by Sanger sequencing of 50 bacterial colonies. In parallel, the same cDNA preparation was analyzed by Q-RT-PCR with *var* domain primers (3-5). The major transcript amplified with DBLα domain primers was extended through the flanking CIDR domain by amplifying and assembling three different fragments. Fragment 1 was amplified by primer varF_dg2 forward and primer DBLα-CIDRα reverse. Fragment 2 was amplified by primer DBLα-CIDRα forward and primer DBLβ12 and DBLβ3/5 reverse. Fragments 1 and 2 were connected by two specific designed primers F_3173-S 5’- GGATATGATTGTACGAAACG - 3’ and R_3173-S 5’- CCATGCTTCATAACATTCAC - 3’ according to the following PCR conditions: 98 ^o^C for 5 min, followed by 40 cycles of 98^o^C for 30 s, 46^o^C for 1 min, 68^o^C for 3 min and final extension at 68^o^C for 7 min. Amplicons were gel-purified and cloned into PCR-Blunt II- TOPO plasmid. At least 5 different colonies per fragment were analyzed by Sanger sequencing. Fragment assembly and alignment was analyzed using Geneious. Sequences were deposited at Genbank with accession numbers MH492721- MH492726.

**Human brain microvascular endothelial cell characterization.** HBMEC monolayer cultures were characterized by flow cytometry under both resting conditions and after 18 h stimulation with 10 ng/ml TNF-α. Cells were lifted with 10 mM EDTA and then rinsed with Hanks balanced salt solution prior to incubation with surface receptor antibodies: EPCR was detected with rat anti-human EPCR 252 mAb (Sigma-Aldrich E6280-200UL, clone RCR-252, 1:40) followed by a goat anti-rat Alexa 488 labeled secondary antibody (ThermoFisher A-11006, 1:400). ICAM-1 was detected by a phycoerythrin (PE)-labeled mouse mAb (Abcam ab19756, 1:25), CD36 was detected by a mouse mAb conjugated to fluorescein isothiocyanate (FITC) (Abcam ab39022, clone TR9, 1:25), and CD31 was detected by a PE-conjugated mouse anti-CD31 mAb (BD Pharmingen mAb, 560983, clone WM59, 1:2.5). Cells were fixed with 2% (vol/vol) paraformaldehyde for 10 min before analysis with a BD LSRII flow cytometer. Protein surface receptor expression was analyzed with FlowJo v10 software.

**Ultrastructural analysis.** For transmission electron microscopy of the IE-vessel wall interaction, 3D microvessels were perfused with IT4var19-IE (2x10^7^ IE/ml in CSC complete media) for 30 min, followed by three 10 min washes by gravity driven flow. 3D brain microvessels were then perfused with modified Karnovsky’s fixative (2% paraformaldehyde, 2.5% glutaraldehyde in 0.2 M cacodylate buffer) overnight before post-fixation in 2% osmium oxide/0.2 M cacodylate buffer and dehydration through graded ethanol series. After embedding dehydrated samples in Epon 812 resin, ultrathin (~70 nm) samples were sliced, and then stained with a combination of uranyl acetate and lead citrate for contrast. Sections were viewed on a JEOL JEM-1400 Transmission Electron Microscope.

**Quantification of cell shape index and mitochondrial fraction in microvessels.** Mitochondrial fraction was calculated from transmission electron micrographs (n = 6) of a resting 3D HBMEC microvessel as the sum of individual mitochondrial areas to total cell area.

**References**

1. Janes JH, Wang CP, Levin-Edens E, Vigan-Womas I, Guillotte M, Melcher M, Mercereau-Puijalon O, Smith JD. 2011. Investigating the host binding signature on the Plasmodium falciparum PfEMP1 protein family. PLoS Pathog 7:e1002032.

2. Soerli J, Barfod L, Lavstsen T, Bernasconi NL, Lanzavecchia A, Hviid L. 2009. Human monoclonal IgG selection of Plasmodium falciparum for the expression of placental malaria-specific variant surface antigens. Parasite Immunol 31:341-346.

3. Lavstsen T, Turner L, Saguti F, Magistrado P, Rask TS, Jespersen JS, Wang CW, Berger SS, Baraka V, Marquard AM, Seguin-Orlando A, Willerslev E, Gilbert MT, Lusingu J, Theander TG. 2012. Plasmodium falciparum erythrocyte membrane protein 1 domain cassettes 8 and 13 are associated with severe malaria in children. Proc Natl Acad Sci U S A 109:E1791-800.

4. Mkumbaye SI, Wang CW, Lyimo E, Jespersen JS, Manjurano A, Mosha J, Kavishe RA, Mwakalinga SB, Minja DT, Lusingu JP, Theander TG, Lavstsen T. 2017. The Severity of Plasmodium falciparum Infection Is Associated with Transcript Levels of var Genes Encoding Endothelial Protein C Receptor-Binding P. falciparum Erythrocyte Membrane Protein 1. Infect Immun 85.

5. Lennartz F, Adams Y, Bengtsson A, Olsen RW, Turner L, Ndam NT, Ecklu-Mensah G, Moussiliou A, Ofori MF, Gamain B, Lusingu JP, Petersen JE, Wang CW, Nunes-Silva S, Jespersen JS, Lau CK, Theander TG, Lavstsen T, Hviid L, Higgins MK, Jensen AT. 2017. Structure-Guided Identification of a Family of Dual Receptor-Binding PfEMP1 that Is Associated with Cerebral Malaria. Cell Host Microbe 21:403-414.
